# Oxidative Dissolution and the Aggregation of Silver Nanoparticles in Drinking and Natural Waters: The Influence of the Medium on the Process Development

**DOI:** 10.3390/toxics12100757

**Published:** 2024-10-18

**Authors:** Vadim A. Ershov, Boris G. Ershov

**Affiliations:** Frumkin Institute of Physical Chemistry and Electrochemistry, Russian Academy of Science, Leninsky Pr. 31-4, 119071 Moscow, Russia; ershov@ipc.rssi.ru

**Keywords:** silver nanoparticles, dissolution, aggregation, natural water, environmental fate, carbonate ions

## Abstract

Currently, there are quite a few data on the ways silver nanoparticles get into the aquatic environment, on their subsequent dissolution in water, and on the release of toxic Ag^+^ ions. Differences in the experimental conditions hinder the determination of the basic regularities of this process. In this study, the stages of oxidative dissolution of AgNPs were studied, starting from the formation of silver hydrosol in deaerated solution, the reaction of silver with oxygen and with drinking and natural waters, the analysis of intermediate species of the oxidized colloidal particles, and the subsequent particle aggregation and precipitation, by optical spectroscopy, DLS, TEM, STEM, and EDX. In the presence of oxygen, silver nanoparticles undergo oxidative dissolution, which gives Ag^+^ ions and results in the subsequent aggregation of nanoparticles. The carbonate hydrosol loses stability when mixed with waters of various origin. This is due to the destruction of the electric double layer, which is caused by an increase in the solution’s ionic strength and the neutralization of the charge of the metal core. The environmental hazard of the silver nanoparticle hydrosol would noticeably change and/or decrease when the nanoparticles get into natural waters because of their fast precipitation and because the major part of released Ag^+^ ions form poorly soluble salts with ions present in water.

## 1. Introduction

Currently, the synthesis and application of nano-sized metal particles is one of the fastest growing areas of nanotechnology. Studies of silver nanoparticles (AgNPs) of various shapes and sizes deserve special mention [1,2,3,4,5,6,7]. Like other nano-sized metals, they have a large surface-area-to-volume ratio, which endows them with unique properties and accounts for their high efficiency. The extensive use of silver nanoparticles in medicine, agriculture, and various fields of science and technology is due to their specific antibacterial and optical characteristics. In particular, they are used in medical textiles, dental implants, therapy, and drug delivery [8,9,10,11,12].

The wide use of materials incorporating silver nanoparticles inevitably leads to silver nanoparticles being present in the environment [13]. Silver may occur in the environment as both ions and nanoparticles and may undergo various physicochemical transformations depending on the particular characteristics of the medium. For example, in the case of a large content of fulvic acids in the aqueous medium, ions can be reduced to form nanoparticles, while in the presence of some salts, silver can form insoluble compounds and pass into bottom sediments [14]. In well-aerated waters, the oxidative dissolution of nanoparticles takes place to give toxic silver ions [15]. It is noteworthy that these processes can occur in parallel, and hence, they are difficult to control.

The uncontrolled spread of silver in the natural environment is dangerous for living organisms and gives rise to environmental hazards. AgNP migration, aggregation, and dissolution in natural water results in their distribution in the habitat and increases their toxic action. The study of these processes and their regularities is needed for an evaluation of the environmental and human health risks and methods to prevent them [16,17,18,19,20,21].

Therefore, it is important to study the impact and influence of silver nanoparticles on environmental components, in particular to determine the limits of tolerance and the resistance of organisms. Currently, there are a lot of data on the ways by which silver nanoparticles get into the aquatic environment, their dissolution in water, and the release of toxic Ag^+^ ions [16,22,23,24,25,26,27]. The dissolution of AgNPs in pure water accompanied by the release of Ag^+^ ions takes place in the presence of air [28,29] and is enhanced by increasing solution acidity [24,29,30,31,32,33]. The Ag nanoparticle size has a pronounced effect on the dissolution. As the size decreases, the dissolution rate increases [28,29,34,35,36,37,38].

AgNP behavior in natural waters is considered in a number of studies [14,15,18,23,28,29,33,34,35,37,38,39,40,41,42,43,44,45,46,47]. As investigation objects, the authors used AgNPs stabilized by citrate ions [29,30,37,44], polyethyleneimine [43], TWEEN-80 [45], PVP [47], and other compounds. Reservoir water [44], river water [46], superficial water [45], and other types of natural water were used in experiments. A considerable influence of the nature of the starting material (type of nanoparticles, stabilizers, etc.) and the water composition and salinity on the efficiency of dissolution and stability of colloidal silver particles was demonstrated.

Differences in experimental conditions using AgNPs of different origin, the particle size and the nature of stabilizer, the water composition and pH, and other factors complicate the identification of basic regularities in the process and its relationship with the environmental conditions. It is unknown which properties and characteristics of nanoparticles and solutions are most significant and control the dissolution of silver. Therefore, despite the considerable progress in the investigation of the water solubility of silver nanoparticles, many questions still remain open and imply the need for further research in this area.

The key goal of the present study is to develop a systemic approach to studying the oxidative dissolution of silver nanoparticles in a hydrosol that is compositionally similar to natural freshwater and contains no compounds used for the synthesis and stabilization that are untypical of drinking or natural water. An important factor is also continuity and the order of investigating the stages of the oxidative dissolution of silver nanoparticles, starting from their production in a deaerated solution. This factor, which has not been considered in previous studies, makes it possible to evaluate the role of the juvenile (unoxidized) metal surface. The subsequent oxidation upon the injection of air accompanied by the release of Ag^+^ ions is considered to determine the stability as a function of oxidation state. The final stage is to study the effect of the composition, рН, and other factors of natural water on the oxidation up to complete aggregation and metal precipitation. This will be performed using AgNPs obtained by the photochemical reduction of silver ions by oxalate ions in a deaerated neutral aqueous solution. The neat silver hydrosol obtained in this way contains virtually only 10 nm nanoparticles stabilized by hydrogen carbonate ions, HCO_3_^−^. The hydrosol pH (6.9–7.1) and HCO_3_^−^ contents (0.5–1.0 × 10^−3^ mol L^−1^) resemble those of some uncontaminated natural freshwaters [48]. The stages of AgNP oxidative dissolution will be studied successively, including the hydrosol formation in a deaerated solution, the interaction of the hydrosol with oxygen, and the analysis of the intermediate forms of the oxidized colloidal particles. The final stage is particle aggregation and precipitation. The nature of AgNPs in solution is investigated in situ by optical spectroscopy using the localized surface plasmon resonance (LSPR) and interband transition (IBT) absorption bands characteristic of AgNPs and by the dynamic light scattering (DLS) of nanoparticles.

## 2. Materials and Methods

### 2.1. Reagents and Solvent

Silver perchlorate monohydrate (AgClO_4_ × H_2_O, 99%, Sigma-Aldrich, Saint Louis, MO, USA) and potassium oxalate monohydrate (K_2_C_2_O_4_ × H_2_O, 99.9%, special purity grade, Reakhim, Moscow, Russia) were used to prepare silver hydrosols.

All reactant solutions and culture media were prepared using doubly distilled deionized water. Deionization was performed on a Vodoley setup with the monitoring of the conductivity of deionized water. Water was purified by being passed through a blend of deionization resins and sorbents. The specific electrical conductivity of deionized water did not exceed 0.20 µS cm^−1^, рН = 6.3–7.2.

### 2.2. Silver Hydrosol Synthesis

A pure silver hydrosol containing only nanoparticles and carbonate ions was prepared by the UV irradiation of a mixture of silver perchlorate AgClO_4_ (source of silver ions) and potassium oxalate K_2_C_2_O_4_ (source of oxalate ions) solutions. The reactant concentrations were (1–3) × 10^−4^ mol L^−1^ for AgClO_4_ and (2–5) × 10^−4^ mol L^−1^ for K_2_C_2_O_4_. The irradiation was carried out in a special reactor equipped with a quartz optical cell with optical path lengths of 5 and 10 mm. The cell volumes were 2 and 4 mL, respectively. A more detailed description of the method was reported previously [49,50].

An Alpha-05 (Melitta, Moscow, Russia) pulse unit with a continuous-spectrum high-power UV xenon lamp was used as the source of UV radiation. The total flux intensity was *I*_UV_ = 6.0 × 10^20^ quanta s^−1^ = 1.0 × 10^−3^ einstein s^−1^. The luminous flux of a xenon lamp covers the entire UV and visible range, resembling most closely the spectrum of sunlight up to 1000 m above sea level.

### 2.3. Determination of the Concentration of Dissolved Silver Ions

The concentration of Ag^+^ ions released upon the dissolution of nanoparticles C_0_[Ag^+^] was determined using the formula

(1) $$C\left[{Ag}^{+}\right]=C_{0}\left[{Ag}^{0}\right]-C_{\tau }\left[{Ag}^{0}\right],$$

where C_0_[Ag^0^] is the concentration of silver ions in the hydrosol at the initial time point, C_τ_[Ag^0^] is the concentration of silver ions in the hydrosol at a particular time point.

The concentrations of silver atoms in hydrosols were determined by the spectrophotometric method described in our earlier publication [51]. The method is based on the IBT absorption intensity of the metal in the UV region. For 5–25 nm particles, the molar extinction coefficient of Ag^0^ atoms in the (0.2–5.0) × 10^−4^ mol^−1^ L^−1^ concentration range was taken to be 3500 ± 100 L mol^−1^ cm^−1^ at λ = 250 nm.

For comparison, the Ag^+^ concentration was also determined by ICP-MS on an Element 2 (Thermo-Finnigan, Bremen, Germany). To separate the ions from the nanoparticles, a silver-containing solution was centrifuged on an Eppendorf 5424 centrifuge (Eppendorf AG, Hamburg, Germany) at 14,600 rpm for 30 min. After centrifuging, optical spectra of the samples were measured to determine the extent of nanoparticle removal. The spectral bands characteristic of silver nanoparticles was absent in the resulting spectra, indicating the complete removal of nanoparticles and the possibility of subsequent determination of silver only as ions. 

### 2.4. Characterization Methods

#### 2.4.1. Optical Spectrophotometry

Optical spectra were measured using a Cary 100 Scan spectrophotometer (Varian, Palo Alto, CA, USA) equipped with a temperature-controlled Peltier cell at 293 K.

#### 2.4.2. Dynamic Light Scattering (DLS)

The hydrodynamic size and the ζ-potential of the resulting colloids were determined on a Delsa Nano C instrument (Beckman Coulter Inc., Brea, CA, USA) at the scattered laser wavelength λ = 658 nm.

#### 2.4.3. Transmission Electron Microscopy

The nanoparticle size distribution was determined using a JEM-2100 transmission electron microscope at an accelerating voltage of 200 kV (JEOL, Akishima, Tokyo, Japan) with a carbon-coated copper substrate (Carbon Type-B 400 mesh, Cu. Ted Pella Inc., Redding, CA, USA). The TEM images were processed using ImageJ 1.8.0 https://imagej.net (accessed on March–August 2024) and Gwyddion 2.66 http://gwyddion.net (accessed on March–August 2024) software. The size distribution histograms were plotted for 100 randomly selected nanoparticles.

#### 2.4.4. Scanning Transmission Electron Microscopy (STEM) and Energy-Dispersive X-Ray Spectroscopy (EDX)

STEM-HAADF micrographs and STEM–EDX elemental mapping images were obtained in Zeiss Libra 200FE (Berlin, Germany).

### 2.5. Types and Compositions of Water

The following types of water were used to study the behavior of silver nanoparticles in water (for the detailed composition, see Table S1):

- *Tap water* taken from the tap in the apartment, Bolshaya Pirogovskaya street, Moscow.
- *Mineral water* Elbrus, bottled, taken from well 452 in the Neitrino settlement, Kabardino, Balkar Republic.
- *Artesian water* from Pestrikovo village, urban district Stupino, Moscow Region, taken at a 74 m depth.
- *Sea water*, bottled, Black Sea Magic, taken from 20 m depth, 5 km away from the coast, near the village settlement Su-Psekh, Krasnodar Territory.

### 2.6. Statistical Processing

All data were obtained at least in triplicate in independent experiments. Statistical analysis was performed with Origin Pro software using Student’s *t*-test.

## 3. Results and Discussion

### 3.1. Nanoparticle Characteristics

**Figure 1:**
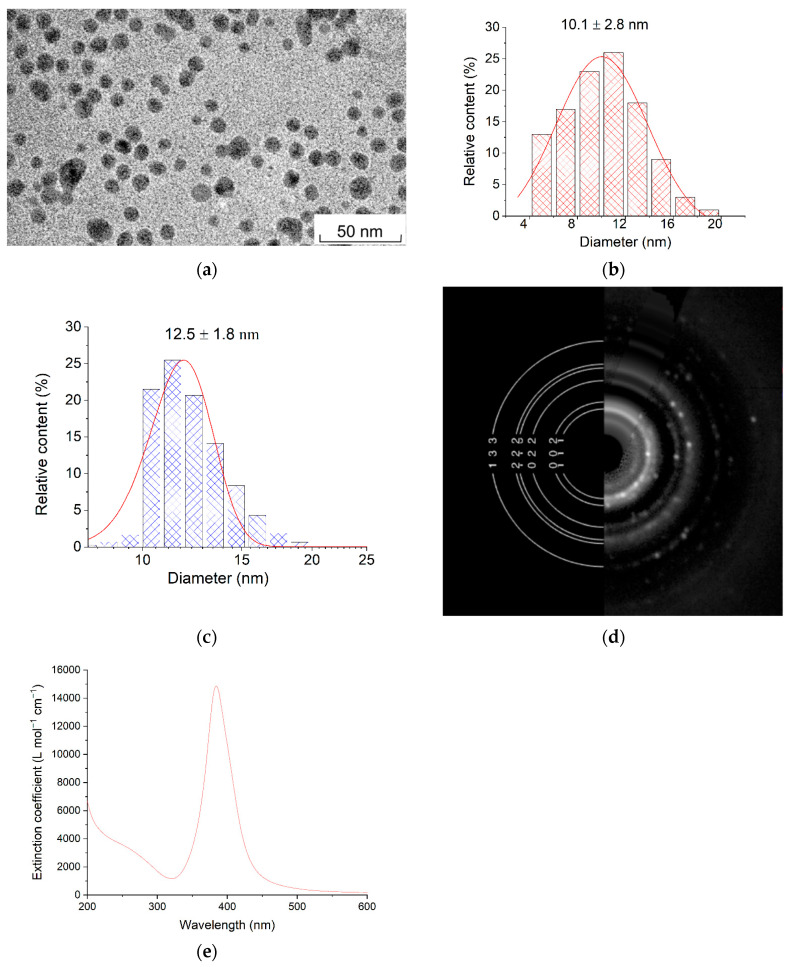
(**a**) TEM image of silver nanoparticles; (**b**,**c**) particle size distribution according to TEM and DLS data, respectively; (**d**) electron diffraction pattern of silver nanoparticles; (**e**) absorption spectrum of silver nanoparticles in a deaerated aqueous solution. Solution: [Ag^+^] = 3 × 10^−4^ mol L^−1^, [$C_{2}O_{4}^{2-}$] = 5 × 10^−4^ mol L^−1^.

The photochemical reduction of deaerated solutions of Ag^+^ ions gave metal nanoparticles (AgNPs). According to the TEM data, these were spherical particles with an average size of (10.1 ± 2.8) nm (Figure 1a,b). The size measured by DLS was somewhat greater, amounting to (12.5 ± 1.8) nm (Figure 1c). This difference is due to the fact that, apart from the metal core detected by SEM, a colloid also has a ligand environment of an electric double layer (EDL). Electron diffraction data revealed structure parameters typical of silver metal and indicated that the nanoparticles were polycrystalline (Figure 1d). The diffraction pattern of the samples obtained in the deaerated solution shows rings 002, 022, 222, and 133 corresponding to the standard (no. 4-0783, The International Centre for Diffraction Data, Newtown Square, PA, USA) of polycrystalline silver. For the samples kept in air, additional rings are observed. The values of interplanar distances correspond to the standard (no. 75-1532, The International Centre for Diffraction Data, Newtown Square, PA, USA) of silver oxide and confirm its formation in small quantities. The absorption spectrum of the spherical silver nanoparticles (Figure 1e) was typical of nano-sized metal [52,53,54,55] and had a narrowly localized surface plasmon resonance (LSPR) band at λ ≈ 400 nm, caused by the collective absorption of free electrons in the metal. The LSPR band overlapped in the UV region (≤320 nm) with the absorption caused by the interband transition (IBT) in the metal atom (4d→5sp).

The pH value of the hydrosol is 7.1. After the complete reduction of Ag^+^ ions, that is, at the final stage of hydrosol formation, the ζ-potential was −110 mV. The negative sign means that the potential-determining layer of the colloid was formed by negatively charged anions (mainly ${HCO}_{3}^{-}$ at pH = 7.1).

It is known [52,53,54,55] that the plasmonic absorption of free electrons in AgNPs is very sensitive to not only the particle size and shape but also the state of particle surface. This is inherent in AgNPs stabilized by carbonate ions, which are used in this study [56,57]. Therefore, changes in the LSPR band’s intensity, shape, and position can be used to consider the state of nanoparticles. Conversely, the particle size and shape and the state of particle surface up to a size of approximately 30 nm have no significant effect on the shape and intensity of the IBT band. The absorbance of silver hydrosol at the IBT wavelength is proportional to the concentration of silver atoms in solution in the form of nanoparticles [51]. The analysis of the optical spectra of AgNPs is useful for investigating particle transformations upon the variation of the composition of solutions and, as shown below, various natural waters.

### 3.2. Oxidative Dissolution and Aggregation of Nanoparticles

#### 3.2.1. Oxidative Dissolution

**Figure 2:**
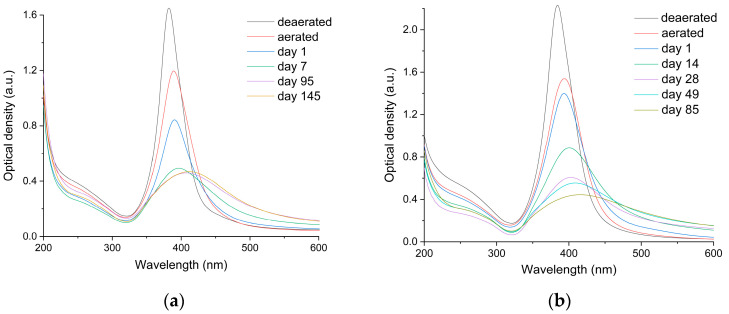
Variation of the absorption spectrum of the deaerated silver hydrosol upon the injection of air and depending on the incubation time. (**a**) Solution: [Ag^0^] = 1 × 10^−4^ mol L^−1^, [$C_{2}O_{4}^{2-}$] = 2 × 10^−4^ mol L^−1^. The optical path length is 10 mm. (**b**) Solution: [Ag^0^] = 3 × 10^−4^ mol L^−1^, [$C_{2}O_{4}^{2-}$] = 5 × 10^−4^ mol L^−1^. The optical path length is 5 mm.

In the absence of air, a silver hydrosol remains stable for many months. This fact is in line with the earlier observation that the dissolution of AgNPs in water with the release of Ag^+^ ions occurs only in the presence of air, i.e., as a result of metal oxidation by oxygen [29,35]. As indicated by the results of the present study, the presence of oxygen initiates metal oxidation with the release of Ag^+^ ions into the solution. This affects the structure of the electric double layer (EDL) of the particle, causes particle restructuring, and changes the stability, and at the final stage, particle aggregation takes place. This process is quite complex and depends on many factors such as particle size and concentration, the nature of the stabilizing layer, the pH, and the presence of various compounds in the solution. Figure 2 depicts the variation of the absorption spectra of deaerated hydrosols depending on the concentrations of silver atoms in the hydrosols after contact with air and on the time of subsequent incubation.

It can be seen that for hydrosols with [Ag^0^] = 1 × 10^−4^ mol L^−1^ and 3 × 10^−4^ mol L^−1^ (Figure 2), the LSPR band is broadened, markedly decreases in intensity, and shifts to shorter wavelengths immediately after the injection of air. The subsequent incubation in air enhances the above changes in the LSPR spectrum. For the hydrosol with [Ag^0^] = 1 × 10^−4^ mol L^−1^ (Figure 2a), a substantial (approximately four-fold) decrease in intensity and broadening of the LSPR band and a shift of the maximum to longer wavelengths up to 410 nm take place after 145 days. Simultaneously, an increase in the long-wavelength absorption up to 800 nm takes place. A similar but markedly more pronounced change in the LSPR absorption takes place for the hydrosol with [Ag^0^] = 3 × 10^−4^ mol L^−1^ (Figure 2b). After 95 days, the LSPR intensity decreases approximately six-fold, and the band shifts to 430 nm. The long-wavelength absorption substantially increases. The effect of oxygen on the IBT band is less pronounced. The injection of air does not change the shape of the band.

However, the intensity of the band sharply decreases after the injection of the air for 24 h (by approximately 40 and 17% of the initial value for [Ag^0^] of 1.0 × 10^−4^ and 3.0 × 10^−4^ mol L^−1^). During the subsequent incubation, the intensity of the IBT band, unlike the LSPR band, decreases only slightly. Recall that the LSPR absorption is sensitive to the change in the nanoparticle state, while the IBT band characterizes the change in the concentration of silver atoms. Therefore, presumably, the oxidative dissolution of nanoparticles upon air injection accompanied by the release of Ag^+^ ions changes the structure of EDL and aggregative stability of the hydrosol. The intensity of the process depends on the initial concentration of nano-sized silver and the concentration of Ag^+^ ions released into the solution.

**Figure 3:**
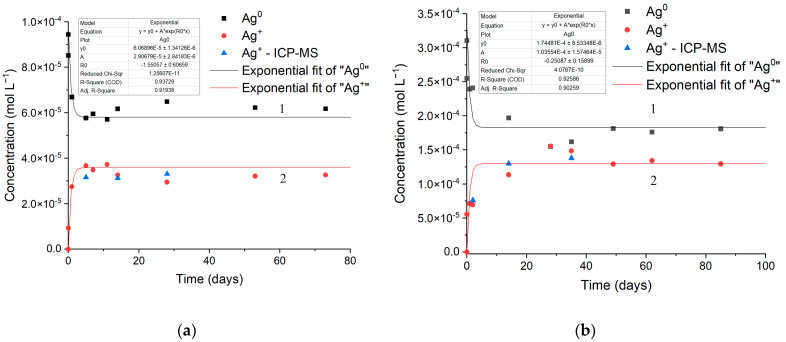
Kinetics of oxidative dissolution of silver nanoparticles and release of Ag^+^ ions vs. time of hydrosol incubation in air: (1) concentration of Ag^0^ atoms; (2) concentration of Ag^+^ ions. The dots are experimental data, the curve is calculation. (**a**) Solution: [Ag^0^] = 1 × 10^−4^ mol L^−1^, [$C_{2}O_{4}^{2-}$] = 2 × 10^−4^ mol L^−1^. The optical path length is 10 mm. (**b**) Solution: [Ag^0^] = 3 × 10^−4^ mol L^−1^, [$C_{2}O_{4}^{2-}$] = 5 × 10^−4^ mol L^−1^.

The kinetics of the oxidative dissolution of silver nanoparticles as a function of incubation time in air (curves 1) and the release of Ag^+^ ions into the solution (curves 2) are shown in Figure 3. The Ag^0^ concentrations were calculated from the IBT band intensity (λ = 250 nm, ε = 3500 L mol^−1^ cm^−1^ [51]). The oxidation of silver atoms results in the formation of Ag^+^ ions. The accumulation of Ag^+^ ions during the oxidation of silver nanoparticles was calculated from the Ag^0^ concentration using relations Δ[Ag^0^]_τ_ = [Ag^0^]_0_ − [Ag^0^]_τ_ and Δ[Ag^+^]_τ_ = Δ[Ag^0^]_τ_ (Figure 3). The Ag^+^ concentrations measured in solution by the independent and direct ICP-MS method are also given. A good agreement can be seen between the data on the release of Ag^+^ ions during the oxidative dissolution of metal nanoparticles calculated from the loss of atoms (decrease in the IBT absorption) and found by direct concentration measurement by ICP-MS. As already noted, there are two stages of hydrosol oxidation upon contact with air. The fast stage ends within approximately 24 h (initial regions of the kinetic plots for Ag^0^ oxidation and Ag^+^ release in Figure 3). This stage is associated with interaction of oxygen with the freshly exposed surface of silver nanoparticles obtained by the reduction of Ag^+^ ions in deaerated aqueous solutions. This interaction leads to the oxidation of Ag^0^ atoms, the formation of the oxide Ag_2_O, and the release of Ag^+^ ions into the solution. The slow stage, observed during storage for 3–5 months, is associated with the subsequent oxidative dissolution of nanoparticles coated by a protective oxide film. The analysis of electron diffraction patterns of the oxidized silver nanoparticles actually demonstrates the presence of Ag_2_O. This silver oxidation stage is more clear-cut for a more concentrated solution (cf. Figure 3a,b).

The kinetics of the dissolution of silver nanoparticles and the release of Ag^+^ ions into the solution, characterized by the decrease in the absorbance of silver atoms, is quite adequately described by first-order kinetics. For the dissolution of nanoparticles:

[Ag^0^]_τ_ = [Ag^0^]_0_ × e^−kτ^, (2)

where [Ag^0^]_0_ and [Ag^0^]_τ_ are the initial and current concentrations of atoms, and k is the reaction rate constant, and for the release of Ag^+^ ions:

[Ag^+^]_τ_ = [Ag^0^]_0_ × (1 − e^−kτ^) (3)

The entire set of experimental kinetic data on the oxidative dissolution of silver nanoparticles is well described within the framework of a first-order reaction. The coefficient of determination (R-square) is 0.94 for 1 × 10^−4^ mol L^−1^ Ag^0^ (Figure 3a) and 0.93 for 3 × 10^−4^ mol L^−1^ Ag^0^ (Figure 3b). That is, there is a good agreement between the kinetic curves and the equations of Ag^0^ disappearance (2) and Ag^+^ formation (3). The rate constant of the oxidative dissolution of silver nanoparticles and release of ions was calculated to be (1.6 ± 0.2) × 10^−3^ min^−1^ or 2.3 day^−1^ for both processes.

This is understandable as the Ag^0^ oxidation and Ag^+^ release kinetics refer to a single process of reactant (Ag^0^) consumption and product (Ag^+^) formation. The fact that the calculated constants are equal within the error confirms the validity of the used model of the oxidative dissolution of AgNPs in water. The oxidative dissolution of silver nanoparticles studied previously [29,30,34,36,37,38] is also adequately described by first-order kinetics. The differences between the kinetic constants of oxidation found in the cited studies and in our work are due to differences in the particle size, the presence of stabilizers, particle pretreatment, and other factors. The fundamental features of our study are that the oxidative dissolution of AgNPs is monitored from the instant the nanoparticles are formed in a deaerated solution and that carbonate ions, intrinsic components of natural waters, are used to stabilize the hydrosol.

#### 3.2.2. Nanoparticle Aggregation

**Figure 4:**
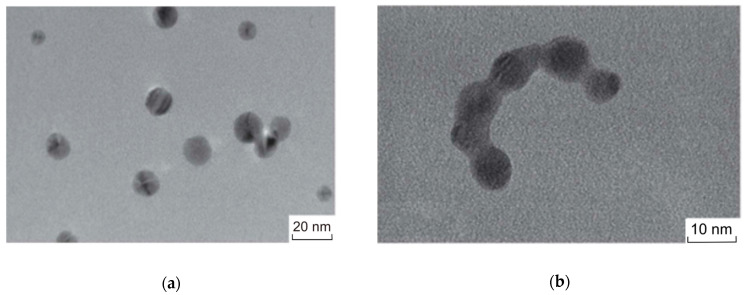
Micrographs of silver nanoparticles (**a**) after they have formed in a deaerated solution and (**b**) after injection of air and incubation for 4 days. Solution: [Ag^+^] = 3 × 10^−4^ mol L^−1^, [$C_{2}O_{4}^{2-}$] = 5 × 10^−4^ mol L^−1^.

The oxidative dissolution of AgNPs is accompanied by the release of Ag^+^ ions into the solution and the formation of ОН^−^ ions. This changes the composition of the medium and, consequently, disturbs the structure of EDL and modifies the hydrosol stability. This conclusion is supported by the observed change in the plasmonic absorption of the hydrosol. As indicated above, on contact with air, the LSPR band shifts to longer wavelengths, and a diffuse declining absorption appears in the 500–800 nm range, because of the formation of large particles and increasing light scattering by nanoparticle agglomerates (Figure 2). Transmission electron microscopy confirms the presence of single spherical nanoparticles uniformly distributed over the substrate surface in a freshly formed silver hydrosol (Figure 4a). After the contact of the hydrosol with air, the formation of agglomerates and chains of bound spherical nanoparticles is observed (Figure 4b).

In the presence of oxygen, the oxidation of nanoparticles results in the release of Ag^+^ ions into the solution, which becomes more pronounced with time. This circumstance significantly affects the state and stability of the silver hydrosol. The appearance of free Ag^+^ ions induces changes in the colloid structure and EDL: the density and the negative charge of the adsorption layer of potential-determining anions increase, which forces it closer to the dense layer of positive counter-ions and leads to the compression of the diffuse layer of colloidal silver particles. The ζ-potential of colloidal particles of the deaerated hydrosol changes from −114 to −67 mV upon the injection of air. The ζ-potential is determined by the thickness of the diffuse colloid layer. The release of Ag^+^ ions leads to a decrease in the ζ-potential and, consequently, the compression of the diffuse layer. The thinner the diffuse layer, the more closely the particles approach one another and the lower the repulsion forces of the particles from other another. The hydrosol stability decreases, which is accompanied by the aggregation of particles and, in the limiting case, precipitation.

#### 3.2.3. Mechanism of Oxidative Dissolution

The oxidative dissolution of silver nanoparticles follows an electrochemical mechanism [58]. It can be represented by two half-reactions occurring in parallel at the electrodes. At the anode, silver atom ionization takes place, and the Ag^+^ ions pass to the solution or form an oxide phase (dissolution) [59]:

2Ag + 2OH^−^ − 2e^−^ → Ag_2_O + H_2_O       E^0^_1_ = 0.342 V (4)

At the cathode, excess electrons interact with dissolved oxygen, which is thus reduced [59]:

O_2_ + 2H_2_O + 4 e^−^ → 4OH^−^       E^0^_2_ = 0.401 V (5)

The clean, unoxidized metal surface is not a source of released Ag^+^ ions [29]. The silver atom ionization [59]

Ag − e^−^ → Ag^+^       E^0^ = 0.799 V (6)

is thermodynamically unfavorable for the dissolution. The dissolution becomes possible owing to the energy benefit caused by the formation of poorly soluble oxide. According to published data [60], the release of Ag^+^ ions into the solution occurs via the dissolution of one or two oxidized monolayers from the particle surface. It was reported [29] that the release of Ag^+^ ions is a co-oxidation process that requires both protons and dissolved O_2_. The silver oxide Ag_2_O is then dissolved as a result of the reaction with protons

Ag_2_O + 2H^+^ → 2Ag^+^ + H_2_O (7)

Thus, being guided by the proposed mechanism of oxidation of silver nanoparticles and summarizing the reactions of Ag_2_O formation and subsequent dissolution, we obtain the following stoichiometric equation for the oxidative dissolution of silver:

1/2O_2_ + 2Ag + 2H^+^ → 2Ag^+^ + H_2_O (8)

At the Ag nanoparticle/aqueous solution interface, EDL is formed, and a potential difference is generated. The electrode potentials determine the degree (proportion of the dissolved metal) and the rate of corrosion. The electromotive force (∆E^0^) for silver oxidation is equal to the difference between the standard potentials of reactions (5) and (4), i.e., ∆E^0^ = (E^0^_2_ − E^0^_1_) = (0.401 V − 0.342 V) = 0.059 V [59]. The positive ∆E^0^ value indicates that metal oxidation is more favorable in the redox process. In the early stage, silver dissolution gives, most likely, the poorly soluble oxide (AgOH/Ag_2_O), which is then slowly dissolved via the reaction with protons. Due to low electromotive force and the formation of a protective oxide film, the oxidation of bulk silver is of low efficiency. However, according to a number of studies [61,62,63,64,65], the standard redox potential of metal nanoparticles differs markedly from that for the bulk metal. It was found that the potential decreases with decreasing particle size [66]. For 10 nm silver nanoparticles, the potential is decreased by approximately −0.100 V. Since here we study nanoparticles of ~10 nm size, we may take this value for the potential. Thus, the potential of reaction (4) should be approximately 0.242 V. Then, the electromotive force ∆E^0^ of the electrochemical oxidation of silver is found to be 0.159 V. The comparison with the value of 0.059 V corresponding to the bulk metal indicates that the nano-sized state of silver should significantly accelerate the oxidative dissolution of the metal in water, and the smaller the particle size, the more pronounced the acceleration. This was observed in quite a few experimental studies [35,36,38]. As shown previously, particle aggregation takes place during the oxidation. Apparently, the increase in the silver particle size and the aggregation are accompanied by a gradual increase in E^0^_1_ to a value characteristic of the bulk metal. This important conclusion follows from the data obtained in the studies [35,38] addressing the size dependence of nanoparticle solubility: an increase in the nanoparticle size and the aggregation are accompanied by a decrease in the dissolution rate until a pseudo-equilibrium is established [66]. Apparently, this is caused, among other factors, by the formation of oxide and other protective compounds on the metal surface. Indeed, it can be seen that the fast oxidative dissolution observed at the initial stage of contact of deaerated hydrosol with air (Figure 3) is replaced by process retardation. The oxidation is correlated with the accelerating aggregation of silver nanoparticles, which, as follows from the above, increases the standard electrode potential and decreases the electromotive force of the electrochemical oxidation of silver. This results in almost the complete termination of the oxidative dissolution of nanoparticles.

The electrochemical model of oxidative dissolution of silver nanoparticles in water and in aqueous solutions [58] establishes the relationship between the particle size and the standard electrode potential, a fundamental characteristic of the particle, and reveals its determining effect on the dissolution kinetics. The smaller the particle size, the lower the potential and the higher the rate of oxidative dissolution in an aqueous medium. The results of the performed studies indicate that during the oxidative dissolution by the electrochemical mechanism, characteristics of silver hydrosol such as the size and state of colloids, the composition of the medium, and the EDL structure drastically change, and finally, a decrease in the stability accompanied by nanoparticle aggregation and even precipitation takes place. The nanoparticle aggregates have potentials and low dissolution rates characteristic of bulk silver. Single small nanoparticles, unlike aggregated structures, determine the Ag^+^ release kinetics.

### 3.3. Nanoparticle Stability in Drinking and Natural Waters

Factors of the medium, i.e., water sources differing in the composition and properties, determine the solubility and stability of silver nanoparticles. The regularities of this influence can be followed while monitoring the stability of hydrosols stabilized by carbonate ions in real drinking and natural waters. We studied the changes in the key hydrosol characteristics (optical absorption, TEM and DLS particle size distribution, ζ-potentials, and other characteristics) induced by hydrosol mixing with an equal volume of natural water. Hydrosols obtained in deaerated solutions were used. After the complete reduction of Ag^+^ ions, the hydrosols were kept in air for 24 h. Then, they were mixed with a specified water at a 1:1 ratio.

#### 3.3.1. Characteristics of Natural Waters

The major ions present in the chosen waters are Ca^2+^, Mg^2+^, and Na^+^ ions; hydrogen carbonate ions; and sulfate, chloride, and bromide ions. The Cl^−^, Br^−^, ${SO}_{4}^{2-}$, and I^−^ ions are specifically adsorbed by silver nanoparticles; i.e., Ag^+^ ions form poorly soluble salts with these anions. Table 1 summarizes the concentrations of the key indifferent and specific ions present in the test waters and the ionic strengths. For the complete composition, see Table S1.

#### 3.3.2. Nanoparticle Stability

A silver hydrosol stabilized by carbonate ions loses stability when mixed with the test waters. An enhancement effect can be observed for waters with higher salinity and ionic strength.

**Figure 5:**
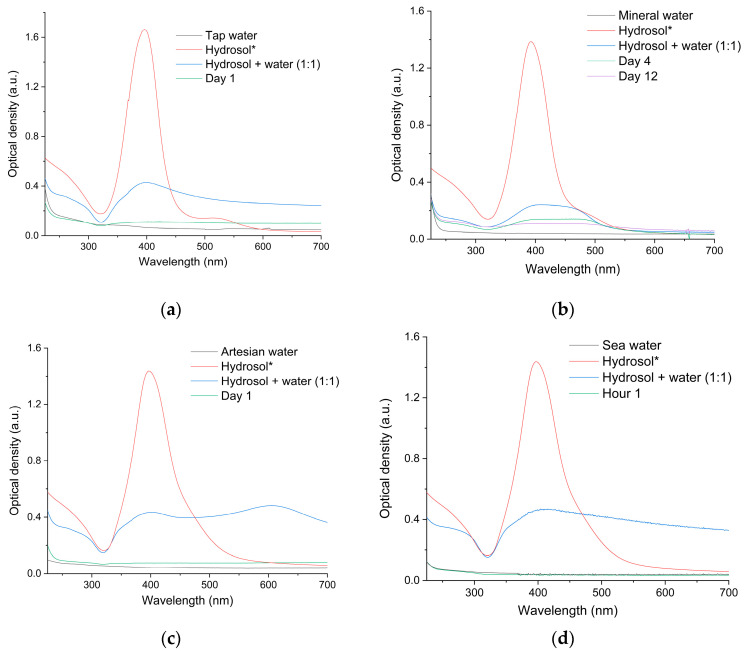
Absorbance of the solution prepared by the mixing of the silver hydrosol (initial [Ag^0^] = 3 × 10^−4^ mol L^−1^) with (**a**) tap water; (**b**) mineral water, (**c**) artesian water, and (**d**) sea water as a function of time. Hydrosol:water = 1:1. The optical path length is 10 mm. * Further dilution by a factor of 2 and a decrease in optical density according to the Beer–Bouguer–Lambert law are taken into account.

Immediately after the mixing of the hydrosol with water taken from the Moscow municipal water pipeline, the structure and shape of the optical absorption spectrum markedly change (Figure 5a).

After mixing, the yellow color characteristic of the hydrosol disappears, and the solution acquires a gray color, indicating the formation of metal particles. In the optical spectrum, the intensity of the optical band at 250 nm caused by IBT decreases, which can be attributed to the oxidation of silver or to the formation of large metal particles and salt precipitate. The LSPR band at 400 nm virtually disappears, and a diffuse broad band with a maximum at ~430 nm and a weak shoulder at ~360 nm appears instead. In the region from 450 nm up to 700 nm, structureless absorption appears, caused by light scattering and reflection from large metal particles with a size comparable with the recording light wavelength [52,53,54,55,67]. The mentioned absorption at ~430 nm with a shoulder at ~360 nm attests to the presence of particles of ~40–50 nm size [68]. The absorbance of a hydrosol/tap water mixture decreases with time, which is caused by increasing solution transparency due to precipitation of metal particles.

Mixing the hydrosol with other types of water, that is, artesian, mineral, and sea water, induces changes in the optical spectrum similar to those observed for tap water, but more pronounced, which attests to fast loss of the stability. Immediately after mixing, the yellow color of the hydrosol disappears. The characteristic structure of the optical absorption of the hydrosol changes. A weak diffuse absorption remains in the LSPR region (Figure 5). The absorbance of the mixtures decreases throughout the day, almost to the value typical of pure water, due to the precipitation of the metal. These facts definitely attest to the aggregation of the initial nanoparticles, considerable enlargement, and precipitation.

The results of the DLS study confirm the enlargement of nanoparticles. The calculated particle size distributions indicate, for example, that immediately after mixing with tap water, the particle size increases from 12 nm, present in the initial hydrosol (Figure 1a), to ~80 nm, and after 24 h, the size reaches ~100 nm. Seven days later, smaller particles (~36 nm) are found in this solution, and they remain in the bulk hydrosol after the precipitation of larger particles.

Similar changes in the particle size, which are even more pronounced, are found for waters with a higher salinity and higher ionic strength. In particular, for the hydrosol with mineral water, the particle size is ~860 nm immediately after mixing, ~150 nm after 24 h, and only ~50 nm after four days. In other words, fast particle aggregation is detected after mixing, and then precipitation of larger particles takes place. As a result, only smaller particles remain in the bulk solution.

**Figure 6:**
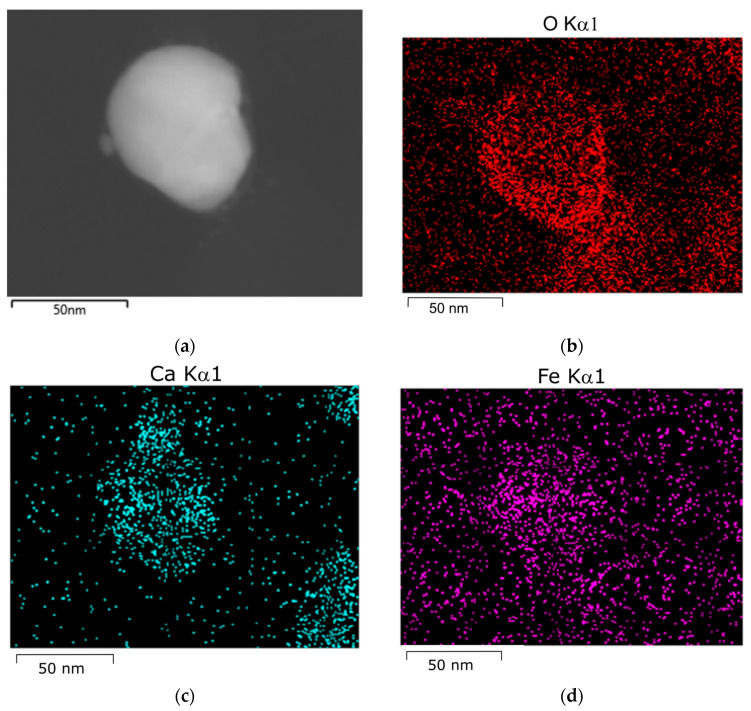

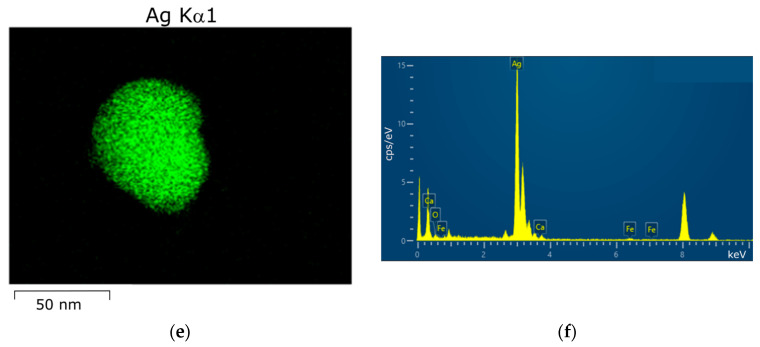
AgNPs 24 h after hydrosol mixing with tap water: (**a**) STEM-HAADF microphotograph; STEM–EDX elemental mapping images: (**b**) O Kα1; (**c**) Ca Kα1; (**d**) Fe Kα1; (**e**) Ag Kα1; (**f**) EDX spectrum of AgNPs kept in tap water for 24 h.

**Figure 7:**
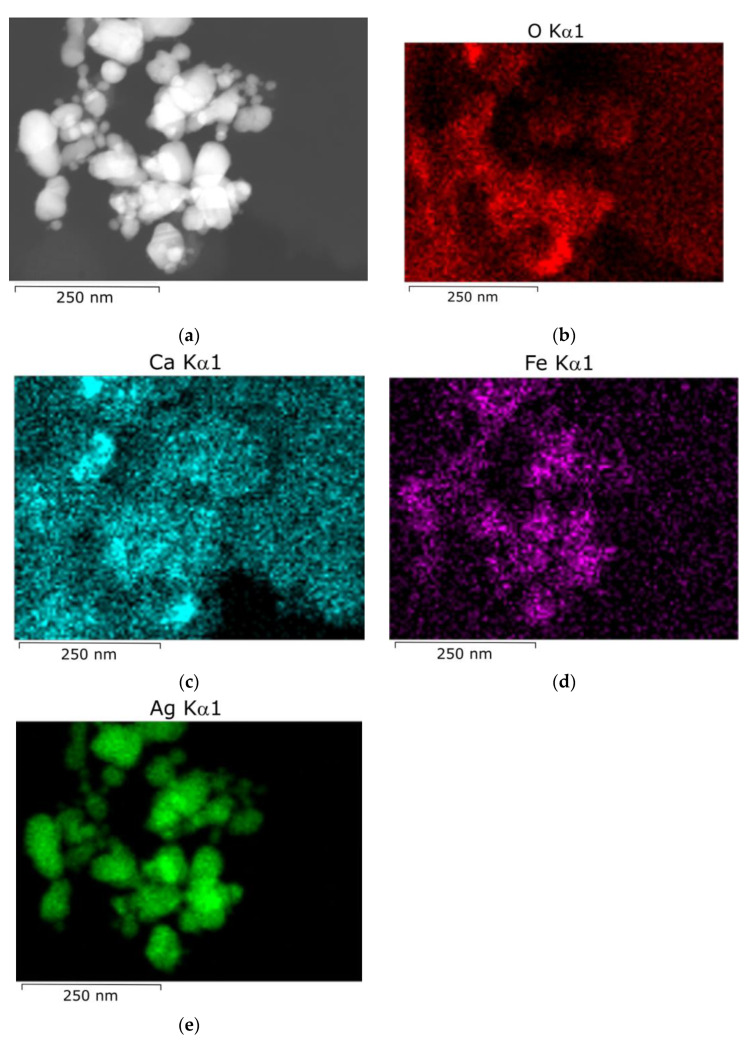
AgNPs 24 h after hydrosol mixing with well water: (**a**) STEM-HAADF microphotograph; STEM–EDX elemental mapping images: (**b**) O Kα1; (**c**) Ca Kα1; (**d**) Fe Kα1; (**e**) Ag Kα1.

TEM data also support the conclusion about the fast aggregation of silver hydrosol nanoparticles upon mixing with drinking and natural waters. After the hydrosol is mixed with tap water, both single spherical nanoparticles of 40–50 nm size (Figure 6) and larger non-spherical particles and particle aggregates were detected. Similar results were obtained for other types of water (Figure 7). They contain aggregates, which represent crowds of closely located particles, which are still separated by interlayers of the medium.

According to elemental analysis of nanoparticles that had been kept for 24 in tap water performed by energy dispersive X-ray spectroscopy (SEM-EDX), Ca and Fe, which occurred in drinking and natural waters, were present on the silver particle surface apart from oxygen (Figure 7).

Thus, mixing hydrosol with drinking and natural waters induces a catastrophic loss of particle stability, resulting in nanoparticle aggregation. The ζ-potential of the initial nanoparticles is −65 mV. After mixing with tap water, this value decreases to −25 mV, while with other types of water, the potential decreases to approximately −17 mV.

The catastrophic effect of natural waters on the stability of silver hydrosols was noted earlier in a number of studies [44,46,47]. For example, the results reported in a study of the effect of Rhine River water on the state of silver hydrosol [46] were similar to those obtained in this study. The TEM and DLS hydrodynamic diameters of AgNPs increased from the initial 30 nm to several hundreds of nanometers 60 min after their addition to the filtered Rhine River water. The ζ-potential of the initial nanoparticles in the hydrosol decreased from −60.8 mV to −14.6 mV after mixing with river water. Analogous data were also reported in another study [44]. It was shown that the aggregative and precipitation stability of citrate-stabilized particles significantly decreased with increasing water salinity. The ζ-potential value changes from −35 mV to −20 mV as the hydrosol is mixed with water, and then it gradually changes to −10 mV by the 7th day. It was also shown that the nanoparticles are aggregated in salt water. The authors noted that the Cl^−^, ${SO}_{4}^{2-}$, and S^2−^ ions, present in large amounts (total salinity of 4.5–16.7 g L^−1^) in the Xi’an Reservoir estuary, increase the ionic strength of the solution, which leads to fast particle precipitation and dissolution.

It was shown [47] that the plasmonic absorption of PVP-stabilized AgNPs considerably decreases in the spring water from Fenghuangling. Inorganic ions, especially divalent Ca^2+^ and Mg^2+^ ions, may be responsible for the AgNP aggregation. In the case of water from the Chaobai River containing high concentrations of dissolved organic matter (DOM), LSPR absorption decreased to a lesser extent, which was attributed to the stabilizing effect of DOM. The most effective hydrosol aggregation was observed for water with a high ionic strength and hardness (Wenyu and Yongding Rivers and Gaobeidian Lake). One more study addressed [44] the stability of citrate-coated AgNPs in natural brackish water collected from six sites with different salinities in the Xinglinwan Reservoir (Xiamen City, southeast China). The results indicated that the AgNP colloids were stable in low-salinity waters, which was mainly due to the stabilizing effect of DOM. However, in high-salinity water, AgNP aggregation and precipitation rapidly took place. It was stated the increase in the cation charge (Mg^2+^ vs. Na^+^) at the same electrolyte concentration (0.01 mol L^−1^) significantly affected electrostatically stabilized AgNPs [26].

The decrease in the ζ-potential on mixing of the hydroѕol with natural waters attests to the disruption of the nanoparticle electric double layer and a decrease in the thickness of the diffuse layer of counter-ions. As a consequence, the mutual repulsion of colloidal particles is weakened, and the hydrosol loses the aggregative stability. The EDL disruption is caused by the change in the composition and the concentrations of ions present in the solution: the initial solution is diluted, and additional ions present in the water are incorporated into EDL. The presence of impurities in water, including ions, increases the ionic strength of the solution, which weakens the stabilizing Coulomb repulsive forces acting between the particles. The particles approach one another and aggregate; i.e., small particles stick together to give larger particles [23]. The Cl^−^, Br^−^, ${SO}_{4}^{2-}$, and also I^−^ ions form poorly soluble salts with Ag^+^ ions. The solubility products of the AgI, AgCl, Ag_3_PO_4_, and Ag_2_SO_4_ salts are 8.5 × 10^−17^, 1.8 × 10^−10^, 8.9 × 10^−17^, and 1.4 × 10^−5^, respectively. Ag_2_S is practically insoluble in water [59]. The precipitation of these salts on the particle surface neutralizes the particle charge, which causes the loss of electrostatic stability. These salts are precipitated, thus purifying the water of Ag^+^ ions. However, the role of such sediments on the habitat can be assessed only by taking into account the nature of their impact on the flora and fauna of water bodies. The possibility that they may become part of food chains that link different groups of organisms such as plants, microorganisms, fungi, and many others cannot be excluded.

#### 3.3.3. Release of Ag^+^ Ions

Mixing of the silver hydrosol with natural waters leads to a loss of stability of the colloidal metal and induces aggregation of particles with subsequent precipitation. It was found that the loss of stability is accompanied by silver oxidation with the release of Ag^+^ ions into the solution. A distinctive feature of the oxidative dissolution is that the release of ions occurs immediately after mixing and during the aggregation of the metal for 24 h. The subsequent incubation of the solution for approximately a month does not virtually induce the formation of additional Ag^+^ ions. This conclusion is in line with the results of a study [44] dealing with the oxidative dissolution of citrate-stabilized silver nanoparticles in natural reservoir water with different salinity. In our experiments, with the initial concentration of carbonate-stabilized nanoparticles [Ag^0^] = 1 × 10^−4^ mol L^−1^, the following concentrations of Ag^+^ ions were found in the bulk solution after mixing with natural water: 3.1 × 10^−4^, 3.6 × 10^−4^, 4.1 × 10^−4^, and 6.8 × 10^−4^ mol L^−1^ for tap, mineral, artesian, and sea water, respectively. The salt content and ionic strength increased in the same series (Table 1). This disrupted the structure of the stabilizing electrical double layer of silver nanoparticles, as indicated by a sharp drop in the ζ-potential. The Br^−^ (3.5 × 10^−4^ mol L^−1^), Cl^−^ (2.81 × 10^−1^ mol L^−1^), and ${SO}_{4}^{2-}$ (9.32 × 10^−3^ mol L^−1^) ions neutralized the surface charge of nanoparticles, which led to nanoparticle aggregation. The aggregation is a catastrophe of a physicochemical system that has occurred in equilibrium. The transition to a more stable state is accompanied by a decrease in the free energy of the system and by energy release. This initiates redox reactions and facilitates the release of Ag^+^ ions. The presence of Cl^−^, Br^−^, I^−^, ${SO}_{4}^{2-}$, and some other ions in the solution makes the formation of poorly soluble salts, AgCl, AgBr, AgI, Ag_2_SO_4_, and others, preferable over reaction (4) giving Ag_2_O. The standard potentials of the electrode half-reactions are as follows [59]:

AgCl + e^−^ → Ag + Cl^−^       E^0^ = 0.222 V (9)

AgBr + e^−^ → Ag + Br^−^       E^0^ = 0.071 V (10)

AgI + e^−^ → Ag + I^−^       E^0^ = −0.152 V (11)

(12) $${\mathrm{Ag}}_{2}{\mathrm{SO}}_{4}+2\;e^{-}\;\to \;2\;\mathrm{Ag}+{SO}_{4}^{2-}\;\;\;\;\;E^{0}=0.654\;V$$

Being guided by the proposed mechanism of oxidation of silver nanoparticles and summarizing the product formation reactions, we obtain the following stoichiometric equation for the oxidative dissolution of silver involving Cl^−^ ions:

1/2O_2_ + 2Ag + 2Cl^−^ + H_2_O → 2AgCl + 2OH^−^ (13)

The presence of Cl^−^, Br^−^, I^−^, ${SO}_{4}^{2-}$, and some other ions in the solution changes the electromotive force of silver oxidation ∆E^0^ = (E^0^_2_ − E^0^_1_), which is equal to the difference between the standard potentials of oxygen reduction (E^0^_2_) and metal oxidation (E^0^_1_). Note that E^0^_1_ (reaction 4) for silver oxidation to Ag_2_O is 0.342 V, while E^0^_2_ (reaction 5) is 0.401 V; hence, ∆E^0^ is 0.059 V. The participation of Cl^−^, Br^−^, I^−^, ${SO}_{4}^{2-}$, and some other ions in the oxidation of silver results in ∆E^0^ being much higher than 0.059 V. Considering the above reactions, the electromotive forces for sliver oxidation ∆E^0^ prove to be 0.179, 0.33, 0.553, and −0.253 V for Cl^−^, Br^−^, I^−^, and ${SO}_{4}^{2-}$, respectively. The ∆E^0^ values are markedly higher than 0.059 V for the Ag_2_O formation; i.e., these reactions are much more thermodynamically favorable. Therefore, in conformity with the series of increasing ∆E^0^ values, one could expect the appropriate increase in the rate and completeness of nanoparticle oxidation. However, as indicated above, this is not the case, and the oxidation proceeds immediately after hydrosol has been mixed with natural waters, and later, it is terminated. This is attributable to the formation of stable poorly soluble salts on the particle surface. The release of silver ions into the solution is determined by the reactions of poorly soluble salt phases with the Cl^−^, Br^−^, I^−^, ${SO}_{4}^{2-}$, and other ions to give soluble complexes such as ${AgCl}_{2}^{-}$, ${AgBr}_{2}^{-}$, and so on. The oxidation of nanoparticles has low efficiency due to the poor solubility of the formed salts. Recall that silver is extracted from ores using the cyanation method, that is, the dissolution of silver in an alkaline solution of NaCN with air access. In this case, the mechanism of metal oxidative dissolution corresponds to the above-described mechanism. However, in this case, soluble Ag(CN)_2_^−^ complex is formed, which provides fast and complete oxidation of the metal [69].

## 4. Conclusions

It was found that silver nanoparticles undergo oxidative dissolution in aqueous solutions in the presence of oxygen. The nanoparticle dissolution and formation of Ag^+^ ions are described by first-order kinetics, with the rate constant being (1.6 ± 0.2) × 10^−3^ min^−1^ in the Ag^0^ concentration range of (1–4) × 10^−4^ mol L^−1^.

The stability of a hydrosol of carbonate-stabilized silver nanoparticles sharply decreases on contact with drinking and natural waters, including tap water, mineral water, artesian water, and sea water. This is manifested in the fact that the initial hydrosol, which remains stable for months, loses stability within one or a few days after mixing with an equal volume of natural water. As this takes place, the nanoparticles clearly tend to aggregate, which ends in the metal precipitation. This is caused by the presence of large amounts of ions, first of all Cl^−^, ${SO}_{4}^{2-}$,Ca^2+^, Mg^2+^, and Na^+^, in the water. These types of water have high ionic strength; this is the main cause for the compression of EDL, which weakens the stabilizing Coulomb repulsion forces acting between particles. As a result, particles approach one another and, eventually, aggregate. The decrease in the hydrosol stability with increasing ionic strength of natural water can be clearly followed.

The foregoing indicates that the environmental hazard of a hydrosol of carbonate-stabilized nanoparticles (apparently, also other nanoparticles with the electrostatic type of stabilization) would be noticeably changed and/or reduced when they get into natural waters due to fast precipitation, with the major part of released Ag^+^ ions forming poorly soluble and insoluble salts with ions present in water.

## Figures and Tables

**Table 1:** Characteristics of the test waters.

Water	Indifferent Ions, 10^−4^ mol L^−1^		Specific Ions, 10^−4^ mol L^−1^		Ionic Strength, mol L^−1^	[Ag^+^], mol L^−1^ *
Tap water	${HCO}_{3}^{-}$	29.6	Cl^−^	8.0	7.5 × 10^−3^	3.1 × 10^−6^
	Ca^2+^	15.0	${SO}_{4}^{2-}$	4.1		
	Mg^2+^	5.9				
	Na^+^	7.8				
Mineral water	${HCO}_{3}^{-}$	25.9	Cl^−^	1.8	3.8 × 10^−3^	3.6 × 10^−6^
	Ca^2+^	8.3	${SO}_{4}^{2-}$	0.26		
	Mg^2+^	3.2				
	Na^+^	1.9				
Artesian water	${HCO}_{3}^{-}$	63.8	Cl^−^	1.8	1.0 × 10^−2^	3.9 × 10^−6^
	${NO}_{3}^{-}$	1.0	${SO}_{4}^{2-}$	1.1		
	Ca^2+^	21.5				
	Mg^2+^	13.2				
	Na^+^	2.4				
Sea water	${HCO}_{3}^{-}$	26.2	Cl^−^	2814.6	3.2 × 10^−1^	6.8 × 10^−6^
	Ca^2+^	55.1	${PO}_{4}^{3-}$	0.9		
	Mg^2+^	253.5	${SO}_{4}^{2-}$	93.2		
	Na^+^	1931.3	I^−^	0.004		
			Br^−^	3.5		

* Concentration of Ag^+^ ions released into the solution after the mixing of the silver hydrosol ([Ag^0^] = 2 × 10^−4^ mol L^−1^) with water (ratio 1:1) after 1 day of waiting.

## Data Availability

The manuscript contains a detailed presentation of the study’s novel findings. Supplementary Materials offer further supporting data. For more information, please contact the corresponding author.

## Supplementary Materials

The following supporting information can be downloaded at https://www.mdpi.com/article/10.3390/toxics12100757/s1: Table S1: Composition and characteristics of waters.
