# Supplementary material for: Oxidative Dissolution and the Aggregation of Silver Nanoparticles in Drinking and Natural Waters: The Influence of the Medium on the Process Development
Source: Toxics. 2024 Oct 18;12(10):757. doi: 10.3390/toxics12100757 (PMC11510811; doi:10.3390/toxics12100757)
Supplement: Supplementary file 1 [file toxics-12-00757-s001.zip › toxics-3220112-supplementary.pdf]

Supplementary Materials

Oxidative Dissolution and Aggregation of Silver Nanoparticles in Drinking and Natural Waters: Influence of the Medium on the Process Development

Vadim Ershov * and Boris Ershov

Frumkin Institute of Physical Chemistry and Electrochemistry, Russian Academy of Science, Leninsky Pr. 31-4,119071 Moscow, Russia; ershov@ipc.rssi.ru
* Correspondence: vadersh@yandex.ru

Composition of waters

The composition and characteristics of waters were determined at the LLC MSULAB laboratory (https://www.msulab.ru/) certified by a state authority of the Russian Federation, Register of Accredited Persons of the Federal Accreditation Service (RussAccreditation). The unique number of the certification record is RA.RU.21OM11.

Table S1. Composition and characteristics of waters.

No.	Parameter, unit of measurement	Tap water	Mineral water	Artesian water	Sea water
	General parameters				
1	pH, pH units	7.2 ± 0.2	8.4 ± 0.2	7.1 ± 0.2	8.1 ± 0.2
2	Hardness (calculated), °degrees	4.17	1.27	6.92	32.6
3	Turbidity (formazin), FTU	< 0.3	< 0.3	< 0.3	< 0.3
4	Permanganate oxidizability / permanganate index, mg L^{-1}	2.4 ± 0.2	< 0.25	< 0.25	n/a
5	Dry residue / Mineralization, mg L^{-1}	270 ± 24	257 ± 35	353 ± 32	18120 ± 300
6	Specific electrical conductivity, μS cm^{-1}	505 ± 25	515 ± 30	599 ± 30	599 ± 30
7	Chromaticity, degrees, Cr-Co scale	4.2 ± 1.3	< 1	< 1	n/a
8	Total alkalinity, mmol L^{-1}	3.25 ± 0.39	2.54 ± 0.41	6.38 ± 0.77	n/a
9	Free alkalinity, mmol L^{-1}	< 0.1	n/a	< 0.1	n/a
	Organoleptic properties				
10	Odor intensity at 20 °C, odor units	0	0	0	0
11	Type of odor at 20 °C	-	-	-	-
	Inorganic compounds				
12	Bromide ions, mg L^{-1}	< 0.05	< 0.05	< 0.05	27.8 ± 0,18
13	Hydrogen carbonate ions, mg L^{-1}	198	158 ± 21	389	176 ± 12
14	Ammonium ions, mg L^{-1}	< 0.05	< 0.05	< 0.05	0.16 ± 0.01
15	Carbonate ions, mg L^{-1}	0	0	0	0
16	Nitrate ions, mg L^{-1}	5.21 ± 0.68	1.09 ± 0.22	6.19 ± 0.80	< 0.1
17	Nitrite ions, mg L^{-1}	0.510 ± 0.066	< 0.02	< 0.1	< 0.1
18	Sulfate ions, mg L^{-1}	39.6 ± 5.1	2.5 ± 0.5	11.4 ± 1.5	895 ± 11
19	Phosphate ions, mg L^{-1}	< 0.1	< 0.1	0.303 ± 0.039	8.4 ± 0.2
20	Fluoride ions, mg L^{-1}	0.179 ± 0.023	0.090 ± 0.25	0.473 ± 0.061	0.207 ± 0.078
21	Chloride ions, mg L^{-1}	28.3 ± 3.7	6.4 ± 1.9	6.39 ± 0.83	9755 ± 500
	Elements (total contents)				
22	Aluminum (total), mg L^{-1}	0.042 ± 0.013	< 0.01	< 0.01	< 0.01

23	Barium (total), mg L^{-1}	0.030 ± 0.009	< 0.05	0.046 ± 0.014	0.074 ± 0.013
24	Iron (total), mg L^{-1}	< 0.05	< 0.03	< 0.05	0.07 ± 0.05
25	Cadmium (total), mg L^{-1}	< 0.0001	< 0.0001	< 0.0001	0.0022 ± 0.0005
26	Potassium (total), mg L^{-1}	3.1 ± 0.5	0.43 ± 0.08	1.34 ± 0.20	1990 ± 30
27	Calcium (total), mg L^{-1}	60 ± 9	33.4 ± 3.6	86 ± 13	221 ± 16
28	Magnesium (total), mg L^{-1}	14.3 ± 2.1	7.7 ± 0.7	32 ± 5	616 ± 27
29	Manganese (total), mg L^{-1}	0.0056 ± 0.0017	< 0.001	< 0.001	0.0084 ± 0.0023
30	Arsenic (total), mg L^{-1}	< 0.005	< 0.005	< 0.005	< 0.005
31	Sodium (total), mg L^{-1}	17.9 ± 2.7	4.4 ± 0.9	5.6 ± 0.8	3048 ± 150
32	Lead (total), mg L^{-1}	< 0.003	< 0.003	< 0.003	< 0.003
33	Strontium (total), mg L^{-1}	0.199 ± 0.040	0.5 ± 0.1	0.21 ± 0.04	2.1 ± 0.12
